# Conservation must capitalise on climate’s moment

**DOI:** 10.1038/s41467-019-13964-y

**Published:** 2020-01-14

**Authors:** Charlie J. Gardner, Matthew J. Struebig, Zoe G. Davies

**Affiliations:** Durrell Institute of Conservation and Ecology, University of Kent, Canterbury, CT2 7NR UK

**Keywords:** Conservation biology, Climate change, Biodiversity

## Abstract

The health of the natural environment has never been a greater concern, but attention to biodiversity loss is being eclipsed by the climate crisis. We argue that conservationists must seize the agenda to put biodiversity at the heart of climate policy.

## Abstract (web summary)

Gardner and colleagues argue that efforts to conserve biodiversity should capitalise on current momentum in the realm of climate change policy.

The environment is finally having its moment. In 2018, Greta Thunberg started her School Strike for Climate^1^, and Extinction Rebellion^2^ launched an uprising against the UK government. Within a few months, both grew into mass civil movements and changed people’s narratives on the environment. Indeed, public concern for the natural world has soared in many industrialised nations, and the environment has never had a higher profile in media or political discourse^3^. This attention is beginning to influence policy at all levels. National governments in Argentina, Canada and France (among others) have declared a climate emergency, and the United Kingdom has enshrined into law a deadline for reaching emissions net neutrality. The call to arms is also being embraced at smaller scales, with half the local authorities in the United Kingdom formally making climate emergency declarations.

However, the discourse is imbalanced. The environmental crisis has two interrelated components, climate breakdown and the loss of biodiversity, and each is as grave and important as the other. Beyond planetary heating, the recent *Global Assessment Report* of the Intergovernmental Science-Policy Platform on Biodiversity and Ecosystem Services (IPBES) found that we also face the extinction of a million species over the coming decades^4^. Yet over the course of this century research funding, research outputs and, in particular, media coverage of climate issues have greatly outstripped those related to biodiversity^5^. In 2016, for example, climate stories featured eight times more than biodiversity ones across the media^6^. This disparity also extends to the political rhetoric, with many institutions declaring a climate emergency, but not a climate and biodiversity emergency.

The climate and biodiversity crises cannot be addressed in isolation, because intact ecosystems are indispensable to efforts to mitigate and adapt to climate change. ‘Natural climate solutions’, including the conservation, restoration and improved use of forests, wetlands and other ecosystems, can provide one-third of the cost-effective mitigation required to deliver the goals of the Paris Agreement^7^. However, at least in forests, this climate regulation service is dependent on the maintenance of intact animal communities^8^. Beyond mitigation, intact ecosystems also offer the most cost-effective defences against climate impacts and, therefore, will be critical to global adaptation efforts^9^.

How society chooses to respond to climate change from this point forward is critical. It is imperative that we avoid the continued degradation of ecosystems, which disrupts their functioning and undermines the contribution they make to climate mitigation and adaptation efforts. For example, we know that the installation of renewable energy infrastructure can be detrimental for sensitive species and habitats^10^, while biofuel production triggers deforestation both directly and indirectly through displacement of food crops^11^. Moreover, human adaptation to climate impacts, such as droughts and destructive storms, will increase pressures on ecosystems as people abandon failing livelihoods and turn to the safety net provided by natural resource exploitation^9^. This phenomenon is likely to be most severely felt in the tropics where biodiversity is concentrated, but where poverty and limited state capacity constrain adaptation options for human populations.

In the headlong rush to implement emergency climate actions, conservationists must ensure that biodiversity is placed at the heart of all future decision-making. Conservationists need to avert the potential threats associated with climate change mitigation and adaptation, and turn them into positive opportunities for biodiversity. One prime example is massive-scale reforestation, which is promoted by the Intergovernmental Panel on Climate Change and numerous international initiatives^12^, and is likely to become a major policy goal globally given that plants are the only effective ‘negative emissions’ technology. Nations such as Brazil, China and India have already made reforestation or forest ‘restoration’ commitments, although these often include plantation forestry using non-native species. However, the natural regeneration of forests is much more effective for carbon sequestration than plantations^13^, and will provide greater biodiversity benefits^14^. Critically, the potential afforestation of non-forest biomes, such as grasslands, risks negative impacts on both biodiversity and emissions, while the focus on forest establishment risks distracting from the more important task of effectively conserving existing carbon-rich ecosystems such as old-growth forests. Therefore, as the world embarks on a strategy of mass reforestation, it is essential that conservationists set the agenda to ensure forest policy is optimised synergistically towards dual climate and biodiversity goals.

Conservationists must reinforce efforts to engage with decision-makers through existing channels, although this has failed to deliver the breadth of policy change needed thus far. We therefore have to make more concerted attempts to engage with those who are pushing the agenda and calling governments and businesses to account, including media, the public and, in particular, the new wave of popular civil movements. The viral growth of the latter, in turn, provides an important lesson in how such engagement can be achieved. The remarkable rise of Greta Thunberg and the youth movement she stimulated can be attributed not to the facts and figures she communicates, but rather to her emotive communication and the impassioned response she inspires. Indeed, experimental psychological research shows that instinctive, emotional thinking sways decision-making more than the deliberative, logical thinking favoured by scientists^15^. Greater scientific understanding does not necessarily lead to increased concern^16^, yet conservation communication strategies all too often remain centred on the transmission of knowledge. Embedding biodiversity issues into mainstream policy will require political will, and this is driven by the public and media. If we are to get the public to care about biodiversity and express their concerns overtly, as they are currently doing for the climate, we may need to become more emotive.

The recent upsurge in concern for the environment is driven largely by climate change, but it presents a unique, and perhaps final, chance to mainstream the conservation of biodiversity. Conservationists must leverage climate’s pre-eminence and capitalise on the opportunities it presents. If we do not, we may not only miss our best opportunity to avoid the extinction of a million species as IPBES warns, but also undermine our prospects for avoiding the worst of the accelerating climate breakdown.
